# Clinical variation in the organization of clinical pathways in esophagogastric cancer, a mixed method multiple case study

**DOI:** 10.1186/s12913-022-07845-2

**Published:** 2022-04-20

**Authors:** J. C. H. B. M. Luijten, P. A. J. Vissers, L. Brom, M. de Bièvre, J. Buijsen, T. Rozema, N. Haj Mohammad, P. van Duijvendijk, E. A. Kouwenhoven, W. J. Eshuis, C. Rosman, P. D. Siersema, H. W. M. van Laarhoven, R. H. A. Verhoeven, G. A. P. Nieuwenhuijzen, M. J. Westerman

**Affiliations:** 1 Department of Research & Development, Netherlands Comprehensive Cancer Organization (IKNL), Godebaldkwartier 419, 3511 DT Utrecht, The Netherlands; 2 Department of Surgery, Radboudumc, Nijmegen, The Netherlands; 3 Department of Gastroenterology, Viecuri Medical Center, Venlo, The Netherlands; 4 Department of Radiation Oncology (Maastro), GROW School for Oncology, Maastricht University Medical Centre+, Maastricht, The Netherlands; 5 Department of Radiation Oncology, Verbeten Insitute, Tilburg, The Netherlands; 6 Department of Medical Oncology, Utrecht UMC, Utrecht University, Utrecht, The Netherlands; 7 Department of Surgery, Gelre Hospital, Apeldoorn, The Netherlands; 8 Department of Surgery, ZGT, Almelo, The Netherlands; 9 Department of Surgery, Cancer Center Amsterdam, Amsterdam UMC, University of Amsterdam, Amsterdam, The Netherlands; 10 Department of Gastroenterology, Radboudumc, Nijmegen, The Netherlands; 11 Department of Medical Oncology, Cancer Center Amsterdam, Amsterdam UMC, University of Amsterdam, Amsterdam, The Netherlands; 12 Department of Surgery, Catharina Hospital, Eindhoven, The Netherlands; 13 Department of Epidemiology and Datascience, Amsterdam UMC, Amsterdam, The Netherlands

**Keywords:** Variation in clinical pathways, Esophageal cancer, Gastric cancer, Cancer medicine

## Abstract

**Background:**

Among esophagogastric cancer patients, the probability of having undergone treatment with curative intent has been shown to vary, depending on the hospital of diagnosis. However, little is known about the factors that contribute to this variation. In this study, we sought to understand the organization of clinical pathways and their association with variation in practice.

**Methods:**

A mixed-method study using quantitative and qualitative data was conducted. Quantitative data were obtained from the Netherlands Cancer Registry (e.g., outpatient clinic consultations and diagnostic procedures). For qualitative data, thematic content analysis was performed using semi-structured interviews (*n* = 30), observations of outpatient clinic consultations (*n* = 26), and multidisciplinary team meetings (MDTM, *n* = 16) in eight hospitals, to assess clinicians’ perspectives regarding the clinical pathways.

**Results:**

Quantitative analyses showed that patients more often underwent surgical consultation prior to the MDTM in hospitals associated with a high probability of receiving treatment with curative intent, but more often consulted with a geriatrician in hospitals associated with a low probability of such treatment. The organization of clinical pathways was analyzed quantitatively at three levels: regional, local, and patient levels. At a regional level, hospitals differed in terms of the number of patients discussed during the MDTM. At the local level, the revision of radiological images and restaging after neoadjuvant treatment varied. At the patient level, some hospitals routinely conduct fitness tests, whereas others estimated the patient’s physical fitness during an outpatient clinic consultation. Few clinicians performed a standard geriatric consultation in older patients to assess their mental fitness and frailty.

**Conclusion:**

Surgical consultation prior to MDTM was more often conducted in hospitals associated with a high probability of receiving treatment with curative intent, whereas a geriatrician was consulted more often in hospitals associated with a low probability of receiving such treatment.

**Supplementary Information:**

The online version contains supplementary material available at 10.1186/s12913-022-07845-2.

## Introduction

Esophageal cancer is the sixth and gastric cancer is the second most common cancer worldwide [1]. Due to the aggressive behavior of these cancers and the associated high mortality rates, patients often have a dismal prognosis [1]. Current clinical practice guidelines state that, depending on the patient’s condition, treatment with curative intent is feasible in patients without distant metastasis or tumor growth into adjacent structures [2–5]. However, in the Netherlands, the probability of a patient undergoing treatment with curative intent for esophageal or gastric cancer varies significantly according to the hospital of diagnosis, and may be associated with the likelihood of survival [6–8]. Patient- and tumor-related factors only partially explain this hospital-associated variability [6–8]. Hence, it has been hypothesized that this variation may well be due to other factors, such as the organization of clinical pathways, multidisciplinary team meetings (MDTM), and compliance with the MDTM recommendation [9–12].

Centralization and regionalization of surgery for esophageal surgery, since 2011, and for gastric cancer, since 2013, has increased, due to a mandated annual volume of at least 20 resections per center across the Netherlands. Due to this centralization, regional upper gastrointestinal (GI) cancer collaborations have been generated between resection and referral centers, with integrated regional clinical pathways and MDTMs [13]. Clinical pathways, which are road-maps describing the sequence and timing of logistic processes, diagnostics, multidisciplinary collaborations, and treatment, have been developed to reduce variability in patient care, as well as to focus more on evidence-based medicine and to prevent treatment delay [14, 15].

Clinical pathways vary between regional networks (collaborating hospital networks established between resection and referral centers) [16, 17]. Generally, studies exploring differences in the organization of clinical pathways among hospitals and among networks are currently lacking. More in-depth information regarding the diagnostics performed, outpatient consultations, and clinician perspectives regarding the organization of their clinical pathways may provide suggestions for optimization of clinical pathways in some hospitals and may elucidate factors explaining the abovementioned clinical variability. Therefore, the aim of this mixed-methods, multiple-case study was to assess differences in the number and type of diagnostics and outpatient clinic visits prior to the MDTM, quantitatively. Second, qualitative methods were used to gain insight into the organization of clinical pathways at the regional, local, and patient levels.

## Methods

### Study design

This study is part of the VARIATE-project (Table 1), which is a mixed-methods, multiple-case study investigating causes of clinical variation in the curative treatment of esophagogastric cancer among hospitals. In the current study, we focused on the organization of clinical pathways, and aimed to describe and understand similarities and differences in the organizations of clinical pathways among hospitals. Quantitative methods were used to gain insight into variations in outpatient clinic consultations and diagnostics used. Semi-structured interviews and observations of MDTMs and outpatient clinic visits were conducted to understand the clinicians’ perspective regarding the organization of their clinical pathways. This study was funded by the Dutch Cancer Society (Project No. 10895).

**Table 1:** The VARIATE project: a mixed methods multiple case study combining qualitative and quantitative research

All patients diagnosed with esophageal and gastric cancer in the Netherlands are registered in the Netherlands Cancer Registry (NCR). Previous multivariable multilevel analyses of potentially curable patients diagnosed in the period 2015–2017 have shown that the probability of receiving treatment with curative intent differed according to the hospital of diagnosis.^1^ Hospitals were divided into three tertiles: low, middle or high probability of undergoing treatment with curative intent using the hospital’s odds ratios based on random intercepts. Patients diagnosed in a hospital with a high probability of receiving treatment with curative intent had a significant better long-term survival.^1^ In order to obtain in-depth information and knowledge of the underlying mechanisms of hospital practice variation in proposing treatment with curative intent the VARIATE project (VariAtion in the cuRatIve treatment of esophAgeal and gasTric cancEr) was developed, which was financed by the Dutch Cancer Society Received treatment with curative intent was defined as endoscopic or surgical resection, initiation of surgery (without resection), definitive chemoradiation (external beam radiotherapy and concurrent chemotherapy; including initiation of definitive chemoradiation). Palliative treatment was defined as: palliative systemic therapy, palliative radiotherapy and best supportive care
**Design:**
The VARIATE project is a mixed methods multiple case study, which combines qualitative and quantitative research. A purposive sample^2^ of eight cases (i.e., hospitals) participated. These hospitals were a representative sample of Dutch hospitals regarding the probability of offering treatment with curative intent, hospital type, size, and geographical location
**Quantitative methods: data collection and analyses**
*Data collection:*
Additional quantitative data for potentially curable patients (cT1-4a or Tx, any cN, cM0) diagnosed in 2015 – 2017 was gathered in 67 hospitals in the Netherlands *(i.e., data was gathered by the NCR regarding diagnostics, the MDTM treatment proposal and outpatient clinic visits)* in order to gain insight in clinical pathways and alterations in MDTM treatment proposal
*Analyses*: Quantitative data was analyzed according to the probability of receiving treatment with curative intent using SAS® version 9.4 (SAS Institute, Cary, North Carolina, USA). A *p*-value below 0.05 was considered statistically significant
**Qualitative methods: data collection and analyses**
*Data collection:*
*Recruitment:* Surgeons or medical oncologists from 11 different hospitals were invited by email. After interest was voiced, JL presented the study during the MDTM of the eight interested hospitals to assess the interest of the multidisciplinary team. All hospitals and team members who saw the presentation wished to participate in the project
The project used an iterative approach for qualitative data collection and analyses. Data collection consisted of: 1. Observations of (Upper-GI specific) MDTMs (2 – 4 MDTMs per hospital) and outpatient clinic visits (minimum of 2 outpatient clinic visits per hospital) 2. Semi-structured interviews (*n* = 30) with clinicians involved in the multidisciplinary care for esophageal and gastric cancer *(i.e., surgeons (S, n* = *8), medical oncologist (MO, n* = *6), radiation oncologist (RO, n* = *5), gastroenterologists (GE, n* = *6) and case managers (CM, n* = *5))* 3. Focus groups with clinicians in order to validate and further enrich the results of their own hospital (*n* = 7) 4. Focus groups with patients diagnosed with potentially curable esophageal or gastric cancer were organized to explore factors related to their treatment choices (*n* = 3: low, middle and high probability hospital)
Based on the analysis of the first 3 hospitals the following decisions regarding the quantitative and qualitative data collection in the further hospitals were made: 1. Depending on the emerging topics from previous interviews the topic list was altered *(more focus on: MDTMs, cases of doubt, shared decision making)* 2. Clinicians in the other five hospitals were selected for interviewing through emergent sampling *(i.e., gastroenterologists who did not treat early carcinomas were not invited for participation, recent new members in multidisciplinary teams were not invited for participation)*
*Analyses:*
Qualitative analyses: Interviews were audio recorded, transcribed per verbatim and summarized (all by JL), and shared with the interviewed clinicians serving as member check. Next, the interviews were reviewed and coded, open coding as described by Strauss and Corbin’s grounded theory approach was used.^3^ The first 11 transcripts were independently coded by two researchers (JL, PV) and discussed until consensus was reached.^4^ The remaining 19 transcripts were coded by JL. Using thematic content analyses emerging themes were found.^5^ Thereafter, through a constant comparison across and within cases (axial coding), relations were searched for and themes were identied.^6^ The core study group (JL, PV, RV, GN) met weekly to discuss analyses, refine the codebook and identify emerging themes. The coding process was facilitated by Atlas ti 8 software
**References**
1. Luijten JCHBM, Vissers PAJ, Lingsma H, van Leeuwen N, Rozema T, Siersema PD, et al. Changes in hospital variation in the probability of receiving treatment with curative intent for esophageal and gastric cancer. Cancer Epidemiol. 2021;71(Pt A):101,897 2. Patton MQ. Qualitative evaluation and research methods. Thousasnd Oaks, CA: Sage. (1990) 3. Strauss AL, & Corbin, J. M.. Basics of qualitative research: Grounded theory procedures and techniques.. Thousand Oaks: Sage Publications. 1990 4. Korstjens I, Moser A. Series: Practical guidance to qualitative research. Part 4: Trustworthiness and publishing. Eur J Gen Pract. 2018;24(1):120–4 5. Green, J., Thorogood, N., Qualitative Methods for Health Research (3th edn.). London: Sage Publications.2013 6. Yin RK. Case study research: Design and methods. Thousand Oaks, CA: Sage. 1994

### Data collection procedures

#### Quantitative research

During the period 2015‒2017, all patients diagnosed with esophageal (including gastroesophageal junction [GEJ]/cardia) and gastric cancer, with a potentially curable tumor stage (cT1-4a, X, any cN, cM0) for which treatment had not yet been initiated, were selected from the nationwide population-based Netherlands Cancer Registry (NCR). Information on patient and tumor characteristics was extracted from medical records by specifically trained NCR data managers.

For this study, in a sample of esophagogastric cancer patients, additional data were collected in a random sample of hospitals associated with a low, middle, and high likelihood of providing treatment with curative intent. A detailed description of the calculation of the probability of treatment with curative intent has been reported previously [8]. These hospitals included academic resection centers (i.e., an university hospital performing resections), resection centers (i.e., a non-university hospital performing resections) and referral centers (i.e., a local hospital solely diagnosing patients and referring patients for esophageal or gastric resections). Additional data were collected on esophageal cancer patients in 38 hospitals and on gastric cancer patients in 68 hospitals. As the incidence of gastric cancer is lower, we included more hospitals (*n* = 68) to reach a representative sample for patients diagnosed with gastric cancer. These data consisted of additional information regarding outpatient clinic consultations with medical oncologists, radiation oncologists, surgeons, and geriatricians prior to the MDTM, as well as the use of endoscopic ultrasound (EUS) and positron emission tomography (PET)‒computed tomography (CT) for restaging prior to the start of treatment. Staging laparoscopy (SL) prior to neoadjuvant treatment was performed in patients diagnosed with gastric cancer.

#### Qualitative research: observations, interviews and focus groups

Eight hospitals (see methods in Table 1) were selected based on hospital type (academic resection hospitals [*n* = 3], regional resection hospitals [*n* = 4], and referring hospital [ *n* = 1]), probability of having undergone treatment with curative intent (low [*n* = 2], low/middle [*n* = 2], and high probability [*n* = 4)), [8] hospital size, and geographical location in the Netherlands, with a view to assess the maximum variation among hospitals. See methods in Table 1 for a detailed description.

From January 2019 to November 2020, observations of MDTMs as well as outpatient clinic visits, interviews, and focus group (FG) discussions with clinicians were conducted. Data were collected and analyzed iteratively. All data were collected by a medical doctor (JL) who was trained to interview organized FGs. All data were analyzed by JL together with two researchers with experience in the field of qualitative research (LB, MW). In the first three hospitals that were visited, upper-GI cancer specialists, including medical oncologists, surgeons, radiation oncologists, gastroenterologists, and case managers (e.g., nurse practitioners and physician assistants) were observed and interviewed. The most important themes of the first three hospitals were determined and discussed by the research team. In the next five hospitals, clinicians were selected for an interview through emergent sampling, i.e., sampling decisions were made during the process of data collection as the study progressed (Table 1) [18]. See for a comprehensive description of the respondents the method section in Table 1.

#### Observations

In total, 16 MDTMs were observed in seven resection hospitals, and 26 outpatient clinic visits were observed in all hospitals. The MDTM observations lasted 1‒1.5 h and included the logistics and organization of clinical pathways, the setting and interaction in the MDTM between participating clinicians and other health professionals, and the process of clinical decision-making. The observations in the outpatient clinics lasted approximately 4 h (each) and focused on logistics, the interaction between the involved clinicians and patients, and the process of treatment decision-making. Field notes were recorded during the observations and were summarized at the end of each observation. Observations and informal conversations were helpful in building a relationship of trust (rapport) and were used as inputs for the interviews.

#### Interviews

A topic list based on the literature (e.g., the organization of healthcare and MDTMs, [19–21] and physicians’ attitudes toward treatment options [22]) was developed (Supplementary Material 1). All semi-structured interviews used a uniform format, first asking the clinician to describe the clinical pathway in their hospital. This was followed by topics such as the organization of the MDTM, shared decision-making, and emerging themes during the interview. During the course of the study, the topic list and interviews became more focused *(*e.g., focusing on restaging, revision of radiological images, protocols, involvement of non-upper-GI disciplines, and pre-habilitation*).* The interviews were performed by one researcher (JL) and had a mean duration of 39 min (range: 25‒56 min). Interviews were audio-recorded, transcribed verbatim, and summarized (JL). A summary of the interviews was sent to each participant to check for correct interpretation, serving as a member check, and was approved by all participants.

#### Focus groups

FG discussions with professionals were conducted in the seven resection hospitals, with three to four clinicians per hospital. In the included referral center, only two clinicians were involved in the clinical pathway of this patient population; hence, this group was too small for an FG. The FGs were organized after the observations and interviews had been conducted. Each FG started with a presentation of the most important results of the observations and interviews, followed by a discussion, during which the clinicians were encouraged to explore, add, or contradict the results of their hospital. FG discussions were held at the hospital (*n* = 3) or by video-conference (*n* = 4) due to the Coronavirus Disease 2019 pandemic, and lasted for an average of 1 h and 30 min. The FG was moderated by the interviewer (JL) and was observed by a second member of the research team (PV or RV). Directly after the FGs, the FG moderator and observer deliberated the results of the FG discussion, and thereafter the audio-recordings were summarized.

### Data analyses

#### Quantitative data analyses

The primary outcome parameters were differences in the proportion of patients seen by medical oncologists, radiation oncologists, surgeons, and geriatricians during the outpatient clinic visit prior to the MDTM (local or regional), and in the diagnostics performed, among the groups defined by the probability of having undergone treatment with curative intent. Treatment with curative intent was defined as endoscopic or surgical resection, initiation of surgery (without resection), and definitive chemoradiation (Table 1). In addition, differences in the proportion of patients seen by a multidisciplinary team during the outpatient clinic visit prior to the MDTM (local or regional), among the groups defined by the probability of having undergone treatment with curative intent, were assessed. “Multidisciplinary” was defined as a medical oncologist, radiation oncologist, and surgeon for an esophageal cancer patient and as a medical oncologist and surgeon for a gastric cancer patient.

Baseline characteristics and patient outcome parameters were assessed as frequencies with percentages according to the probability of having undergone treatment with curative intent and were compared using chi-square or Fisher’s exact tests, as appropriate. Outpatient clinical consults prior to the MDTM, the use of PET-CT, EUS, SL (for gastric cancer only), and restaging after neoadjuvant treatment were analyzed for all potentially curable patients. The use of a geriatric assessment was analyzed by stratifying patients into different age groups (i.e., < 70 years, 70–74, 75–80, > 80 years).

#### Qualitative data analyses

The analyses section of Table 1 describes a detailed description of the qualitative data analyses. Field notes of observations focusing on the organization of clinical pathways and logistics, transcripts of the interviews on clinical pathways, and summaries of the clinician FG discussions were used for a thematic content analysis (see Table 1 for more details). For each hospital, a similar thematic map summarizing each theme and subtheme per clinician was created to identify overarching themes. Through constant comparison within and across cases, associations were searched and potentially deviant cases were identified [23]. Preliminary conclusions resulting from the data described in the thematic map were thoroughly discussed by the research team (JL, PV, RV, GN, MW). The topics of the discussion were the themes and interrelations between the codes and themes described in the thematic map. The themes were thereafter discussed with an expert in the field of clinical pathways (JvH).

### Ethics

The Medical Research Ethics Committees United (MEC-U, Nieuwegein, The Netherlands) confirmed that ethical approval was not required for the qualitative part of this study (file number: W.18.166), as it was not considered as research under the Medical Research Act (WMO). This study was conducted in accordance with the principles of the Declaration of Helsinki. The participating hospitals approved the study. Written informed consent was obtained from all the participants prior to the interviews. The audio recordings and transcripts were stored pseudonymized for a minimum of 10 years on the secured network of IKNL, with only the core research team members having access.

According to the Central Committee on Research involving Human Subjects in The Hague, the Netherlands, the quantitative part of study does not require approval from an ethics committee in the Netherlands. All quantitative data collected by the NCR were de-identified and pseudonymized. The privacy review board of the NCR approved the access and use of the NCR data.

## Results

### Quantitative results: variation in practice

Table 2 displays the patient characteristics according to the probability of having undergone curative treatment in the hospital of diagnosis. There were no significantly different distributions of patient and tumor characteristics among the groups with low, middle, or high probability of having undergone treatment with curative intent for esophageal cancer (Table 2). Significant differences were observed in the number of comorbidities *(0 comorbidities: 33%, 39%, 40%, 1 comorbidities: 37%, 34%, 28%, 2 or more comorbidities: 27%, 23%, 28%, unknown 4%, 5%, 4% for a low, middle or high probability respectively, p* = *0.01)* and the ECOG performance status *(ECOG 0 and 1: 51%, 43%, 49%, ECOG 2: 7%, 7%, 9%, ECOG 3 and 4: 6%, 3%, 2%, Unknown ECOG 35%, 46%, 39%, for a low, middle or high probability respectively, p* = *0.001)* for gastric cancer (Table 2). Patients were more often consulted by surgeons prior to the MDTM during an outpatient clinic visit in hospitals associated with a high probability of having undergone treatment with curative intent for both esophageal *(54% vs. 63%, for a low vs. high probability, p* < *0.01)* and gastric cancer *(61% vs. 69%, for a low vs high probability, p* = *0.01).* Esophageal cancer patients were more often had a multidisciplinary consultation prior to the MDTM in hospitals associated with a high probability of having undergone treatment with curative intent. Patients with esophageal cancer were more often consulted by geriatricians prior to the MDTM in hospitals associated with a low probability of having undergone treatment with curative intent *(7% vs. 3%, for a low vs. high probability, p* < *0.01)*. Similarly, patients with gastric cancer were less often consulted by geriatricians in hospitals associated with a medium probability of having undergone treatment with curative intent *(11%, 6%, 12%, for a low, middle or high probability respectively, p* < *0.01 middle vs. high)*. No differences were observed in the use of EUS, PET, SL, and restaging PET-CT when comparing the low-, medium-, and high-probability hospitals (Table 3).

**Table 2:** Patient and tumor characteristics of patients with esophageal or gastric cancer according to hospital with low, middle and high probability of receiving treatment with curative intent

	Esophageal cancer							Gastric cancer						
	probability							probability						
	low		middle		high		*p* value	low		middle		high		*p* value
	N	%	N	%	N	%								
ALL	477	100%	548	100%	625	100%		507	100%	327	100%	545	100%	
Sex							0.75							0.53
Female	136	29%	147	27%	179	29%		199	39%	122	37%	224	41%	
Male	341	71%	401	73%	446	71%		308	61%	205	63%	321	59%	
Age							0.09							0.72
< 60	100	21%	83	15%	111	18%		76	15%	46	14%	71	13%	
60—74	239	50%	287	52%	340	54%		169	33%	122	37%	195	36%	
≥ 75	138	29%	178	32%	174	28%		262	52%	159	49%	279	51%	
cT Classification							0.59							0.03
cT1	28	6%	35	6%	34	5%		21	4%	12	4%	29	5%	
cT2	133	28%	143	26%	197	32%		202	40%	117	36%	190	35%	
cT3	239	50%	273	50%	305	49%		130	26%	66	20%	117	21%	
cT4	6	1%	10	2%	10	2%		16	3%	20	6%	19	3%	
cTX	71	15%	87	16%	79	13%		138	27%	112	34%	190	35%	
cN Classification							0.22							0.7
cN0	176	37%	211	39%	272	44%		275	54%	182	56%	317	58%	
cN +	260	55%	288	53%	303	48%		161	32%	99	30%	151	28%	
cNX	41	9%	49	9%	50	8%		71	14%	46	14%	77	14%	
Histology							0.07							0.475
Adenocarcinoma	348	73%	386	70%	477	76%		491	97%	321	98%	532	98%	
Squamous cell carcinoma	123	26%	145	26%	134	21%		NA		NA		NA		
Not otherwise specified	6	1%	17	3%	14	2%		16	3%	6	2%	13	2%	
Number of Comorbidities							0.83							0.01
0 comorbidities	192	40%	213	39%	259	41%		166	33%	127	39%	219	40%	
1 comorbidity	151	32%	184	34%	204	33%		187	37%	111	34%	153	28%	
2 or more	116	24%	117	21%	141	23%		135	27%	74	23%	150	28%	
unknown	18	4%	34	6%	21	3%		19	4%	15	5%	23	4%	
ECOG performance status							0.53							0.001
ECOG 0 and 1	319	67%	368	67%	415	66%		260	51%	141	43%	267	49%	
ECOG 2	39	8%	53	10%	43	7%		37	7%	24	7%	51	9%	
ECOG 3 and 4	16	3%	16	3%	17	3%		31	6%	10	3%	12	2%	
Unknown ECOG	103	22%	111	20%	150	24%		179	35%	152	46%	215	39%

**Table 3:** Quantitative outcome parameters oesophageal and gastric cancer according to the probability of receiving treatment with curative intent

	**Oesophageal cancer probabiliy**								**Gastric cancer probability**							
	**Low**		**Middle**		**High**		***p* value low versus high**	***p* value middle versus high**	**Low**		**Middle**		**High**		***p* value low versus high**	***p* value middle versus high**
**Total**	**477**	**100%**	**548**	**100%**	**625**	**100%**			**516**		**333**		**560**			
**PET-CT**	**347**	**73%**	**425**	**78%**	**458**	**73%**	**0.84**	**0.09**	**103**	**20%**	**44**	**13%**	**114**	**21%**	**0.81**	** < 0.01**
**EUS**	**229**	**49%**	**266**	**49%**	**293**	**47%**	**0.71**	**0.57**	**70**	**14%**	**37**	**11%**	**69**	**13%**	**0.58**	**0.56**
**DLS**	**NA**		**NA**		**NA**				**91**	**18%**	**66**	**20%**	**94**	**17%**	**0.77**	**0.28**
**Prior to MDTM**																
**Radiation oncologist**	**233**	**47%**	**263**	**48%**	**329**	**53%**	**0.053**	**0.11**	**9**	**2%**	**7**	**2%**	**12**	**2%**	**0.62**	**0.95**
**Medical oncologist**	**305**	**64%**	**324**	**59%**	**401**	**64%**	**0.94**	**0.08**	**293**	**58%**	**189**	**58%**	**316**	**58%**	**0.95**	**0.96**
**Surgeon**	**259**	**54%**	**301**	**55%**	**393**	**63%**	** < 0.01**	** < 0.01**	**311**	**61%**	**198**	**61%**	**374**	**69%**	**0.01**	**0.02**
**Multidisciplinary**^b^	**193**	**40%**	**250**	**46%**	**310**	**50%**	**0.003**	**0.173**	**249**	**495**	**155**	**47%**	**266**	**49%**	**0.92**	**0.69**
**Geriatrician prior to MDTM**	**33**	**7%**	**24**	**4%**	**20**	**3%**	** < 0.01**	**0.29**	**56**	**11%**	**20**	**6%**	**66**	**12%**	**0.59**	** < 0.01**
**Geriatrician prior to MDTM according to age groups**																
** < 70 years**	**7**	**58%**	**1**	**8%**	**4**	**33%**	0.10*	**0.22**	**2**	**29%**	**1**	**14%**	**4**	**57%**	**0.26**	0.27*
**70—74**	**14**	**52%**	**8**	**20%**	**5**	**19%**	** < 0.01**	**0.41**	**11**	**48%**	**4**	**17%**	**8**	**35%**	**0.37**	0.23*
**75—80**	**10**	**36%**	**10**	**36%**	**8**	**29%**	**0.31**	**0.58**	**20**	**39%**	**5**	**10%**	**26**	**51%**	**0.55**	**0.02**
** > 80**	**2**	**20%**	**5**	**50%**	**3**	**30%**	0.35*	0.24*	**23**	**38%**	**10**	**16%**	**28**	**46%**	**0.52**	**0.1**
**Number of patients treated with neoadjuvant therapy**	**184**		**263**		**316**				**171**		**125**		**192**			
**Restaging CT**	**36**	**20%**	**71**	**27%**	**82**	**26%**	** < 0.01**	**0.7**	**115**	**67%**	**79**	**63%**	**115**	**60%**	**0.58**	**0.61**
**Restaging PET-CT**	**98**	**53%**	**88**	**33%**	**145**	**46%**	**0.45**	** < 0.01**	**5**	**3%**	**9**	**7%**	**10**	**5%**	**0.12**	**0.36**

^*^Fishers exact test

^b^Multidisciplinary for esophageal cancer is defined as: radiation oncologist, medical oncologist and surgeon. For gastric cancer multidisciplinary is defined as medical oncologist and surgeon

### Qualitative results: variation in the organization of clinical pathways

**Fig. 1:**
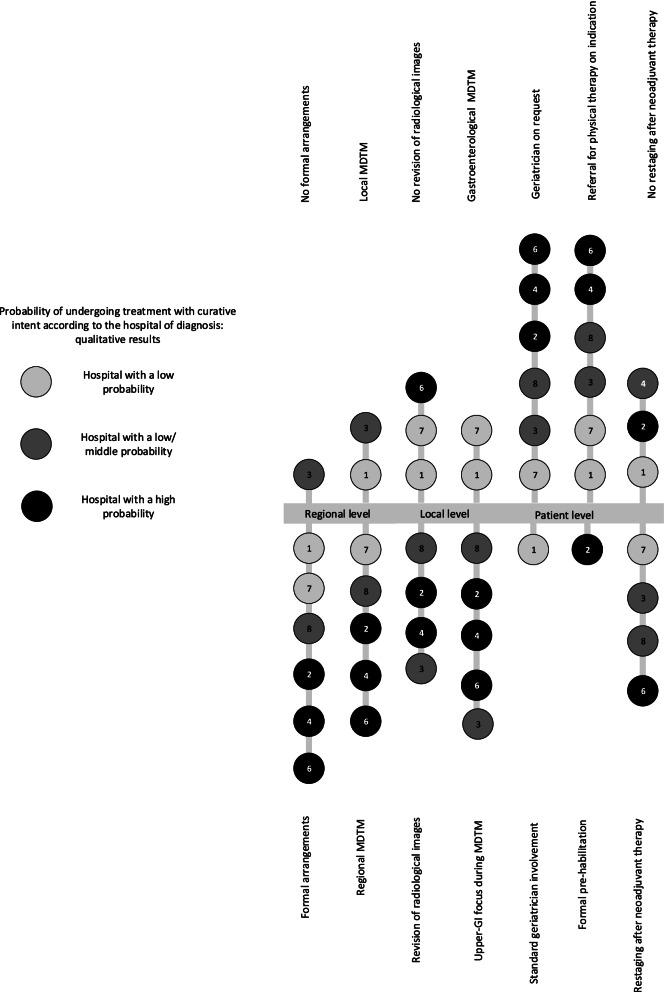
Parts of an integrated clinical pathway according to the hospital of diagnosis on regional, local and patient level. Based on the observations all hospitals are displayed in this figure. Each included hospital is represented by the form of a circle including a hospital number. In addition the probability groups are represented by different shades of gray. The referring hospital is not represented in this figure

The results based on observations and interviews were described in three themes related to selection of the clinical pathway on different levels: regional, local, and patient levels (Table 4). The regional level describes arrangements for the referral of patients, centralization of care, and organization of diagnostics and communication. The local level provides more insight into patient information and diagnostics, organization of the MDTM, and setting and organization of the MDTM. The patient level provides information that is necessary for treatment decision-making and clinical examination of the patient’s physical and cognitive functioning, and its organization differed between hospitals. Figure 1 displays the variation among hospitals in terms of the organization of integrated pathways and shows that formal arrangements, regional MDTMs, and upper-GI MDTMs were more often observed in hospitals associated with a high probability of having undergone treatment with curative intent. The categories presented in bold in Table 4 are described in more detail below.

**Table 4:** Variation in the organization of clinical pathways on regional, local, and patient level

Theme	Subtheme	Category	Summary
Regional level	Arrangements for referral of patients	• Arrangements versus no arrangements	Most hospitals were part of a regional network or collaboration in which agreements regarding referral were established. Other hospitals had no agreements at all
		**Referral of patients to a resection center** • All patients *(including all tumor stages and postoperative patients)* • Selection of patients *(e.g., refraining from referral of those with high age, metastasized disease, decreased physical functioning)* • Second opinion	Differences across hospitals were observed regarding patient discussion during the MDTM. Some hospitals discussed all patients, while others who discussed only a selection of patients
	Centralization of care	• **Knowledge** • Increased experience due to higher exposure to Upper-GI cases in resection centers • Decreased exposure in referral hospitals *(e.g., premature or overdue referral, not aware of possibilities and impossibilities)*	More knowledge in resection centers and potential knowledge depletion in referral centers due to differences in exposure
	Organization of diagnostics and treatment	• Diagnostics conducted in the referral center and/or resection center	Arrangements regarding conducting diagnostics in the referral or resection center differed and, in most hospitals, this was described in their clinical pathways
		**Quality of radiological images** • Sufficient versus insufficient • Protocols versus no protocols	The quality of the radiological images differed per referring hospital. At times if quality was insufficient, repeated radiological imaging was necessary. Some hospitals established protocols to improve the quality of radiological images
		• Arrangements about the hospital of treatment • Referral versus resection center ▪ Neoadjuvant treatment ▪ Adjuvant treatment ▪ Follow-up	Hospitals’ agreements differed considerably regarding the location (resection- and/or referring hospital) of neoadjuvant therapy and follow-up or the location of treatment of complications
	Communication	• Consultation between centers • Difficult cases	Some resection hospitals organized overarching MDTMs with other resection centers in order to discuss difficult cases
		**Coordination** • Case manager is key figure	The case manager in the resection center was an invaluable asset in the care pathway by facilitating communication between and within centers
Local level	Patient information and diagnostics	• **Standard templates with patient information for MDTM**	All hospitals used standard templates for patient discussion during an MDTM; however sometimes information was lacking, resulting in postponement of patient presetationto the next MDTM
		• **Revision of radiological images by dedicated in-house radiologist** *(e.g., finding of additional metastases in lymph nodes or resulting in conversion of cM1 to cM0 staging)*	Standard revision of radiological images of referred patients by an in-house upper-GI radiologist prior to the MDTM could lead to additional findings, such as the identification of additional metastatic locations or resectability, influencing the treatment plan in either direction
		• Patient representation by clinician during the MDTM	In some hospitals, the patient is represented by a clinician (e.g., clinician from referral or resection center), while in others, the patient discussion is based on the standard templates
	Organization of MDTM	• MDTM Preparation • Patient summary • Patient’s seen during outpatient clinic visit prior to MDTM • **Triage** (*e.g., monitor if all data is accessible prior to MDTM)*	Some hospitals conducted triage prior to the MDTM, reviewing and verifying whether all necessary diagnostic modalities were performed and all information was obtained and available, enhancing discussion, resulting in a more efficient workflow during the MDTM. If certain modalities had not been implemented, these examinations were ordered after triage so that patient information would be complete during the MDTM
	Setting and buildup of the MDTM	• Dealing with missing clinical information *(e.g., complete versus incomplete information)* • Time-related aspects of the MDTM *(e.g., attendees being late, last-minute applications)*	Complete information available during the MDTM makes treatment decision-making (TDM) feasible. Last-minute application for discussion of patients in the MDTM leads to incomplete information. If attendees are late to attend the MDTM, knowledge remains lacking until all involved medical specialists are present to participate in the MDTM. The maximum time an MDTM should last was mentioned to be 1.5 h, since at the end fatigue set in. Because fatigue sets in toward the end of the MDTM, some hospitals discussed the new cases at the beginning of the MDTM, followed by recurring cases
		• **Discussed tumor types** *(e.g., solely upper-GI leads to better focus, gastroenterological cancer patients)*	In the majority of the hospitals only oncologic upper-GI patients were discussed during the MDTM, whereas, in some hospitals, other gastroenterological cancers were also discussed
		**• Attendees** *(e.g., academic center, dedicated team, referring clinicians, live versus teleconference)*	Clinicians may attend the MDTM live or by teleconference. In some non-academic resection hospitals an academic resection hospital was present during the MDTM and in some hospitals the referring clinicians were not present during the MDTM
Patient level	Standardization of clinical examinations	• Histological confirmation of potential metastases • All centers aim for histological confirmation—centers differ in their action if the results are inconclusive	Hospitals differed on histological confirmation of all potential metastatic disease: some always used histological confirmation, and others used it depending on whether it was important for the decision and according to the reliability of the radiological characteristics
		• **Patient’s physical functioning** • Fitness test versus medical history and clinical examination	The patient’s physical functioning was assessed differently. Some hospitals conducted fitness tests *(CPAP, lung function, ECG)*, whereas others made an estimation of patient’s physical fitness based on the medical history and clinical examination
		• **Restaging after neoadjuvant treatment** • Standard • No restaging • High-risk patients for interval metastasis	Half of the hospitals performed restaging by PET-CT or CT after neoadjuvant therapy, leading to avoidance of unnecessary surgery in patients diagnosed with interval metastasis
	Clinical examination of the patient’s physical and cognitive functioning	• **Involving non-upper-GI specialist** *(e.g., frailty estimation)* • Standard arrangements versus based on clinical assessment	Some hospitals consulted non-upper-GI specialists, such as anesthesiologists, prior to TDM. A geriatric assessment was standard in some hospitals in patients above a certain age, to determine mental fitness and frailty, while others only consulted a geriatrician if deemed necessary
		• **Pre-habilitation of physical functioning** • Boosting of physical functioning versus less invasive treatment choice • Active Pre-habilitation programs versus advice/referral	Some hospitals offered a formal pre-habilitation program, including physical therapy and the consultation of a dietician, to improve physical fitness, whereas others only advised patients to improve their physical fitness

Categories in bold are discussed in the result section of this article, *TDM* Treatment decision-making, *MDTM* multidisciplinary team meeting

### Regional level

The integrated clinical pathways of most regional networks describe critical actions and decision points in a patient’s medical care, in terms of the pathway of referral, diagnostics, and treatment. Seven hospitals were part of a tumor-specific regional network in which agreements regarding referral, location, and type of diagnostics and treatment were established, whereas one hospital had no formal agreements. In two resection centers, the MDTMs were local, meaning that referring hospitals did not participate in the MDTM. In the other five resection hospitals, the referring hospitals joined the MDTM, physically or by video-conference.

#### Arrangements for referral

Most clinicians stated that most of the potentially curable patients diagnosed with esophagogastric cancer in their network were discussed during an MDTM. In some hospitals, all diagnosed patients, including early cancer and postoperative patients, were discussed, whereas in other hospitals, patients diagnosed with early cancer, older patients with metastatic disease, or fragile patients with a severely limited physical functioning were not discussed. Some clinicians mentioned that they felt that there was no added value in discussing all patients, regardless of their tumor stage, whereas others felt that discussing all patients is important, since referring clinicians might not always be familiar with all treatment options, and thus multidisciplinary discussion with an expert center is important:*“I think that there are physicians that do not expect additional treatment advice from the MDTM; however I think that that is not always a correct assumption.”* [Medical oncologist 2, High probability (i.e., working at a high probability hospital [8]].

#### Centralization of care

Some clinicians mentioned that, due to centralization, exposure to patients diagnosed with esophageal and gastric cancer in referral centers decreased. Thus, all cases should be evaluated by experts, as explained by a surgeon: *“Each patient, regardless of the radiological conclusion… should be discussed centrally or regionally, so that each patient has the maximal probability of optimal treatment advice”* [Surgeon 2, High probability]. Most clinicians felt that, due to the decreased number of cases in the referring center, knowledge in these centers decreased, which could potentially negatively affect willingness to refer patients: *“I believe that, due to the decreasing knowledge regarding a specific disease, physicians may tend to refer patients either prematurely or too late.”* [Medical oncologists 1, Low probability].

#### Organization of diagnostics

All hospitals aimed to collect complete patient information prior to the MDTM, although this was not always possible. Some clinicians mentioned that the quality of the radiological images differed across referring hospitals, and sometimes a repeat radiological examination was necessary. One clinician stated that his/her hospital had developed a protocol to overcome this lack of quality (i.e., including CT- and radiological protocols in their hospital): *“Everything, including the CT- and radiological protocols, should be standardized regionally. In the past, CT scans obtained elsewhere were determined to be of insufficient quality, necessitating radiological examinations to be repeated.”* [Gastroenterologist 4, High probability].

#### Communication

Most clinicians emphasized that, in general, the case manager in the expert center was an invaluable asset in the clinical pathway, who facilitated communication and coordination between and within centers, as well as conducting outpatient clinic visits in most expert centers.

### Local level

#### Patient information and diagnostics

Predefined checklists for recording information regarding the patient’s history, tumor stage, and in a few hospitals, patients’ treatment preferences, were used in all hospitals, to facilitate patient discussion during an MDTM. However, most clinicians explained that patient information and diagnostic information were sometimes incomplete, which complicated decision-making during the MDTM. Hospitals differed in terms of whether or not the patient was presented by a clinician who had seen the patient during an outpatient clinic visit prior to the MDTM. In most hospitals, the patient was referred to another hospital during the MDTM. More detailed knowledge of the patient was considered to improve the quality of the discussion during the MDTM: *“Regarding the history, the social situation, and the patient’s resilience, it is very important that the physician who knows the patient discusses that patient during the meeting, but this is not always the case”* [Medical oncologist 2, High probability]. In one referral hospital, standard consultation of referred patients with a surgeon is completed as standard prior to the MDTM, whereas a few other referral hospitals tried to see as many patients as possible prior to the MDTM. However, other interviewees argued about whether conducting a consult prior to the MDTM added value, since it was their opinion that the consultant of the expert center should assess whether or not the MDTM proposal was a feasible treatment option for that specific patient, *after* the MDMT.

Four referral hospitals always revised all radiological images by an in-house upper GI radiologist prior to MDTM. For most clinicians in these hospitals, this was important since this regularly led to additional findings: *“There are cases where additional metastases were found in initially M0 patients, which makes the revision of radiological images in the referral hospital of the utmost importance.”* [Radiation oncologist 3, Low/Middle probability]. Others explained that they did not conduct standard revision of radiological images because they felt that this would lead to a high workload, without added value. Some clinicians thought that revision of radiological images was unnecessary because the expertise of the referring center’s radiologists was sufficient.

#### Organization of the MDTM

Two referral hospitals (both with a low/middle probability) checked all information before the MDTM in order to identify whether all necessary patient information was available, resulting in a higher quality discussion during the MDTM: *“The supporting staff and the radiologist perform triage in order to ensure that all information is complete, that all necessary imaging has been conducted, that the pathology slides have been requested and revised, and as soon as all imaging is available, they evaluate whether new imaging is needed, if imaging should be repeated, or if the quality of imaging is adequate for a good discussion”* [Case manager 3, Low/Middle probability]. During triage in these two hospitals, it became apparent that certain examinations were not performed. These evaluations were ordered prior to the MDTM to ensure that all necessary information would be available during the MDTM; however, this sometimes resulted in postponing the discussion of the patient by 1 week. These steps led to the availability of complete patient information during the MDTM, which was reported to improve discussion and to result in a more efficient workflow. It was also explained that, whenever the number of patients was too high during an MDTM discussion, the focus of those present decreased and discussions became rushed toward the end of the MDTM.

#### Setting and structure of the MDTM

Most referral hospitals only discussed patients diagnosed with esophageal or gastric cancers during the MDTM. Two referral hospitals discussed all gastrointestinal cancer types during the MDTM. The hospitals in which only upper-GI cancers were discussed felt that this led to a better focus during discussion, as explained by a surgeon: “*We separated the colorectal, upper-GI, and hepatobiliary meetings. I have noticed a quality change in that the upper-GI cancer meeting has since been highly focused.”* [Surgeon 6, High probability].

MDTMs were attended live or by video-conference. In two referral hospitals, only clinicians of the resection hospital were present during the MDTM, as opposed to MDTMs in which all referring hospitals simultaneously participated during the MDTM (*n* = 4), or the alternating attendance of referral hospitals with video-conference during the MDTM (*n* = 1). In one non-academic resection hospital, an academic resection hospital was present during the MDTMs, which they felt resulted in proposal of more invasive treatment options: *“Since the academic center has joined our meeting, neoadjuvant treatment options are more frequently suggested, regardless of whether the cases are potentially curable or not; we have become less conservative.”* [Radiation oncologist, 7, Low probability].

### Patient level

#### Standardization of clinical examinations

Some hospitals routinely conduct fitness tests, such as bicycle ergometry or spirometry, whereas others estimated the patient’s physical fitness during an outpatient clinic consultation.

In four hospitals (*n* = 8), restaging after neoadjuvant therapy was standard and was performed with PET-CT or CT, leading to avoidance of unnecessary surgery in patients diagnosed with interval metastases, as opposed to three hospitals in which restaging was not performed: *“We started with standard restaging as of 2018. We diagnosed a number of postoperative patients with metastatic disease within a short period of time after surgery. At the same time, the results of the PRESANO trial were published describing the occurrence of interval metastasis”* [Surgeon 7, Low probability].

#### Clinical examination of the patient’s physical and cognitive functioning

Few clinicians performed a standard geriatric consultation in older patients to assess their mental fitness and frailty. Sometimes, the treatment plan during the MDTM was changed toward either a curative or palliative direction according to the advice of the geriatrician, as explained by a surgeon: *“If the geriatrician mentions that an important decrease in quality of life is to be expected, which we think is an important factor, their opinion would argue against resection”* [Surgeon 1, Low probability]. Others found that a standard consultation was not valuable, and only consulted a geriatrician when in doubt, to determine whether the expected decrease in quality of life might be unacceptable, and to assess the risk of delirium. The maximum age to perform surgery was regarded differently by different clinicians; some followed the patient’s calendar age, whereas others estimated the patient’s biological age during the outpatient clinic visit.

Some clinicians felt that other medical specialists, such as a cardiologist, pulmonologist, and anesthesiologist needed to be consulted, if this could potentially influence the patient’s probability of undergoing surgery: *“There are many patients that are not operable based on their history, but if you assess them together, and involve the anesthesiologist, they turn out to be operable after all”* [Surgeon 4, High probability].

Some clinicians mentioned that they offered a formal pre-habilitation program, including physical therapy and consultation by a dietician to improve physical fitness. Most hospitals, however, regarded advice to the patient on improving physical functioning or referral to a physical therapist as sufficient. One surgeon explained that the patient’s physical functioning could improve due to formal pre-habilitation, making surgery feasible in patients who might otherwise not be operable prior to pre-habilitation: *“In my experience, if the patient’s outcomes* (regarding fitness tests) *are bad, […] and there are a lot of people whose physical functioning is insufficient, it surprises me how many patients we pull through”* [Surgeon 2, High probability].

## Discussion

In our investigation into the organization of clinical pathways and their association with variation in practice, we found that quantitative factors associated with the probability of undergoing curative treatment prior to MDTM included consultation of surgeons and geriatricians in hospitals with a high or low probability of having undergone treatment with curative intent, respectively. Most of the hospitals included in this study were part of a regional network. The hospitals’ organization of care differed in terms of referral of patients to an MDTM, whether radiological images were revised, patients were restaged after neoadjuvant therapy, the patient’s physical functioning was assessed, and how the MDTM was organized. Formal arrangements between referral and resection centers, participation in regional MDTMs, organization of upper-GI MDTMs, and routinely conducted fitness tests were more often observed in hospitals with a high probability of having undergone treatment with curative intent.

### Regional level

MDTMs are defined as periodical meetings between physicians with different medical expertise. The Dutch SONCOS norms state that 90% of patients diagnosed with a malignancy should be discussed during an MDTM, with the possibility to consult an expert center [13]. However, the result of the current study demonstrated that in some referral hospitals, all patients diagnosed with esophageal or gastric cancer were discussed during the MDTM, whereas in other referral hospitals, only patients who were deemed potentially curable by the referring center were discussed. In accordance with our results, several studies have reported regional and hospital practice variations regarding the number and type of patients discussed during an MDTM. Patients were discussed less frequently if they had a higher age or had metastasized disease [24–26]. Discussion during an MDMT could lead to changes in the primary proposed treatment plan derived by the multidisciplinary discussion, [27] and seems to be associated with survival in head and neck cancers [28], as well as in esophageal cancer. [25]. In addition, referring clinicians might not always be familiar with all treatment options, latest- and emerging evidence, as well as access to clinical trials. Therefore it is an important component of review at specialized hospitals, and hence, we propose that all patients, regardless of their tumor stage and physical fitness, should be discussed during a tumor-specific MDTM.

### Local level

Triage is a method that intends to identify and prioritize patients with critical time-sensitive care needs [29, 30]. Conducting triage prior to the MDTM and protocolizing radiological modalities have been suggested to enhance efficiency during MDTM. Triage prior to an MDTM intends to check whether all necessary information is available prior to the MDTM. The current study clearly demonstrated that triage is perceived to be helpful in identifying and correcting critical aspects necessary for meaningful discussions during MDTM. In addition, as mentioned during the interviews and reported in previous studies, standardized triage processes have a positive effect on efficiency and productivity in teams [31, 32]. Because the case load during most MDTMs is high, it can be hypothesized that triage prior to an MDTM could lead to a more efficient work flow, resulting in less frequent re-discussion of patients during the MDTM. Nevertheless, due to triage, a delay in patient discussion during an MDTM might occur, which may be a disadvantage. Additionally, some hospitals have protocolized radiological modalities and have implemented these in their regional clinical pathways with referral centers, which resulted in a decrease in the necessity to repeat radiological images, thus leading to a reduction in costs, as well as patient burden with repeated diagnostics and delays to diagnosis, staging and treatment initiation.

Furthermore, differences among hospitals regarding routine revision of radiological images of referred patients were observed. A previous study had suggested that both quality of CT scan and radiologist experience are associated with a higher likelihood of a correct diagnosis [33]. It was reported that CT examination by an expert radiologist resulted in a three times more frequent correct diagnosis of distant metastasis [33]. In addition, a recent study in head and neck cancer patients concluded that re-review of radiological images by radiological experts resulted in a change in treatment strategy in 11% of cases [27]. Since in the current study, revision of radiological images was mentioned to lead to changes in the treatment proposal, depending on whether or not metastasis were found, and given that this was most often observed in hospitals with a middle and high probability of having undergone treatment with curative intent, this might contribute to practice variation.

In addition, the qualitative and quantitative results of the present study showed differences among hospitals regarding the performance of restaging after neoadjuvant treatment. In some hospitals with a low probability of having undergone treatment with curative intent, this was performed in all patients, whereas other hospitals did not perform restaging in their patients. Interestingly, previous studies found that in 8‒11% of esophageal cancer patients treated with neoadjuvant chemoradiation, new metastatic lesions were detected during PET-CT restaging [34, 35]. Since restaging was reported to lead to alterations in the treatment plan, variation among hospitals in terms of performing restaging might affect the probability of undergoing surgery.

Nevertheless, the aforementioned identified factors describing variation in clinical pathways among hospitals are highly specific. These factors are not yet described in clinical practice guidelines, and hence may lead to variation in clinical pathways among hospitals.

### Patient level

The quantitative results of the present study demonstrated that, in hospitals with a high probability of having undergone treatment with curative intent, patients were more often seen by a surgeon prior to MDTM. On the other hand, in hospitals with a low probability of having undergone treatment with curative intent, patients were more often seen by a geriatrician prior to the MDTM, which also became apparent during the interviews. In esophageal cancer, patient and tumor characteristics did not differ among the low-, middle-, and high-probability groups. Thus, it can be hypothesized that the implementation of these consultations in the clinical care path differed among hospitals. This might change the treatment plan as advised by the MDTM and result in a variation in the probability of having undergone treatment with curative intent. Hence, it can be speculated that, in borderline cases (in terms of frailty, age, and comorbidities), a surgical or geriatric consultation prior to MDTM might be of added value.

As aging is a process involving gradual loss of physiological reserves, chronological age may differ significantly from biological age, and treatment decisions should therefore not be solely based on chronological age [36, 37]. Nevertheless, based on the qualitative results in the current study, clinicians often regarded the maximum age at which to perform surgery differently (biological versus calendar age), potentially leading to fewer surgical treatment decisions in older patients. In addition, multimorbidity and frailty are common in older patients undergoing cancer surgery, and are associated with worse postoperative outcomes [38, 39]. A recent study on colorectal cancer concluded that a geriatric consultation may be useful in clinical decision-making in older patients. It frequently resulted in changes in the treatment plan and in further optimization of the patient’s health status prior to treatment [40]. Nevertheless, the assessment of an older patient’s physical fitness should not only be assessed by a geriatrician. In order to make the assessment as complete as possible, other professionals from other disciplines, such as dieticians and physical therapists, are also often involved in the assessment of a patient’s function and the possible improvement of their performance status in most hospitals.

The qualitative data showed that clinicians had different opinions regarding the patient’s physical functioning and the importance of a formal pre-habilitation program. Some clinicians felt that, due to pre-habilitation, more patients were considered operable who might not have been considered operable prior to pre-habilitation, and that a formal pre-habilitation program was essential in this respect. In contrast, others believed that referral to a physical therapist was sufficient. Pre-habilitation has been suggested to reduce surgical complications, facilitate the acceptance of other necessary treatments and accelerate recovery when prescribed prior to therapy [41–44]. Additional support during pre-habilitation might be important to improve motivation and overcome barriers to exercise, improving a patient’s physical function and performance status [45].

### Strengths and limitations

The main strength of this study was the combination of quantitative and qualitative research, which provides a broad and in-depth understanding of the differences in the organization of clinical pathways among hospitals, resulting in variation in hospital practices. The reliability and validity of the data were increased due to data triangulation (i.e., using multiple data sources to develop a comprehensive understanding) and member checks (i.e., participant validation) [46, 47]. Nevertheless, there are some limitations that should be considered when interpreting the results, as all observations and interviews were carried out by a single researcher, and thus researcher bias might have occurred. However, peer debriefing was conducted during the period of data collection and analyses, facilitating reflection, which can be considered a strength of this study. The increased involvement of a geriatrician in hospitals with a low probability of having undergone treatment with curative intent should be interpreted with caution, since the assessment of an older patient’s physical fitness is not solely assessed by a geriatrician, and other disciplines are involved. The current study assessed factors that potentially contributed to hospital practice variation. However, based on the results of the current study, it remains unknown whether a low or high probability of undergoing curative treatment is favorable for patients in terms of progression-free survival and quality of life. In addition, for the quantitative analyses data from the period 2015–2017 was used, since this was the most recent data available from the NCR. However, this might be considered as a limitation since clinical pathways might change over time. Nonetheless, based on the interviews the majority of the included hospitals did not implement major changes after that time period. Future studies should assess whether hospital practice variation results in different levels of quality of life experienced by patients. Finally, a proportion of clinicians who treat esophageal- or gastric cancer within the Netherlands was included in this study. The aim of qualitative research is to achieve a representative sample of the population one is investigating, and therefore, it is not necessary to include all clinicians of a specific country or niche, this should however be taken in consideration when interpretating the results.

## Conclusion

Clinical pathways among hospitals treating patients with esophageal or gastric cancer vary, and may impact cancer care. Triage prior to the MDTM, revision of radiological images coming from the referring centers, and pre-habilitation might lead to further improvements in the clinical pathways of patients diagnosed with upper GI cancer. Surgical consultation prior to MDTM was more often performed in hospitals with a high probability of having undergone treatment with curative intent, whereas assessment by a geriatrician was more often completed in hospitals with a low probability of offering such treatment.

## Supplementary Information

**Additional file 1.**

## Data Availability

The data underlying this article cannot be shared publicly due to for the privacy of individuals that participated in the study. The data that support the findings of this study are available from Netherlands Comprehensive Cancer Organization (IKNL) but restrictions apply to the availability of these data, which were used under license for the current study, and so are not publicly available. Data are however available from the authors upon reasonable request and with permission of Netherlands Comprehensive Cancer Organization (IKNL).

## Abbreviations

MDTM: Multi-disciplinary team meetings
GI: Gastrointestinal
FG: Focus group
GEJ: Gastroesophageal junction
NCR: Netherlands Cancer Registry
EUS: Endoscopic ultrasound
PET: Positron emission tomography
CT: Computed tomography
SL: Staging laparoscopy
