# Supplementary material for: Clinical variation in the organization of clinical pathways in esophagogastric cancer, a mixed method multiple case study
Source: BMC Health Serv Res. 2022 Apr 20;22:527. doi: 10.1186/s12913-022-07845-2 (PMC9022421; doi:10.1186/s12913-022-07845-2)
Supplement: Supplementary file 1 — Additional file 1. [file 12913_2022_7845_MOESM1_ESM.docx]

**Supplementary 1: Interview guide**

Introduction of interview

**Initial topic list**

**Organizational context**

Considerations in care provided (patients notes as a reminder):

- Organization of clinical pathway
- Patient discussion (MDTM & with colleagues)
- Are all patients discussed in a MDTM

**Collaboration in region**

- Logistics
- Financial aspects
- Political problems
- Referral
- Process
- Collaboration between centers

**Treatment decision-making**:

- such as: receival, process and influences on treatment

**Knowledge**

- Physician/ hospital
- Conferences
- Centralization and knowledge drainage

**Physician’s preferences**

- Recent positive or negative experience with sort of treatment
- Observed referral difference between colleagues

**Patient related factors**

- Treatment options
- Shared decision making
- Cases of doubt
- Referral

**Considerations in treatment decisions**
